# Chemical composition, seasonal variation and antiaging activities of essential oil from *Callistemon subulatus* leaves growing in Egypt

**DOI:** 10.1080/14756366.2023.2224944

**Published:** 2023-06-27

**Authors:** Omyma Rabie, Heba A. S. El-Nashar, Taghreed A. Majrashi, Tarfah Al‐Warhi, Mahmoud A. El Hassab, Wagdy M. Eldehna, Nada M. Mostafa

**Affiliations:** a Department of Pharmacognosy, Faculty of Pharmacy, Ain Shams University, Abbassia, Cairo, Egypt; b Department of Pharmacognosy, College of Pharmacy, King Khalid University, Asir, Saudi Arabia; c Department of Chemistry, College of Science, Princess Nourah bint Abdulrahman University, Riyadh, Saudi Arabia; d Department of Medicinal Chemistry, Faculty of Pharmacy, King Salman International University (KSIU), Egypt; e Department of Pharmaceutical Chemistry, Faculty of Pharmacy, Kafrelsheikh University, Kafrelsheikh, Egypt

**Keywords:** *Callistemon subulatus*, seasonal variation, Elastase, acetylcholinesterase, molecular docking

## Abstract

*Callistemon* is an aromatic genus of flowering plants belonging to family Myrtaceae. The essential oils of *C. subulatus* leaves were collected in four seasons and analyzed using GC/MS. The oils demonstrated monoterpenes as the predominant class. Eucalyptol was the main component in all seasons; summer (66.87%), autumn (58.33%), winter (46.74%) and spring (44.63%), followed by *α*-pinene; spring (31.41%), winter (28.69%), summer (26.34%) and autumn (24.68%). Winter oil, the highest yield (0.53 mL/100g), was further investigated for its inhibitory activity against enzymes associated with ageing; elastase and acetylcholinesterase. It remarkably inhibited elastase and acetylcholinesterase with IC_50_ values of 1.05 and 0.20 µg/ml, respectively. A molecular docking study was conducted for the major oil components on the active sites of target enzymes. Eucalyptol revealed the best binding affinity for both enzymes. *C. subualtus* oil could be used as supplement for management of ageing disorders like skin wrinkles and dementia.

## GRAPHICAL ABSTRACT

**Figure:**
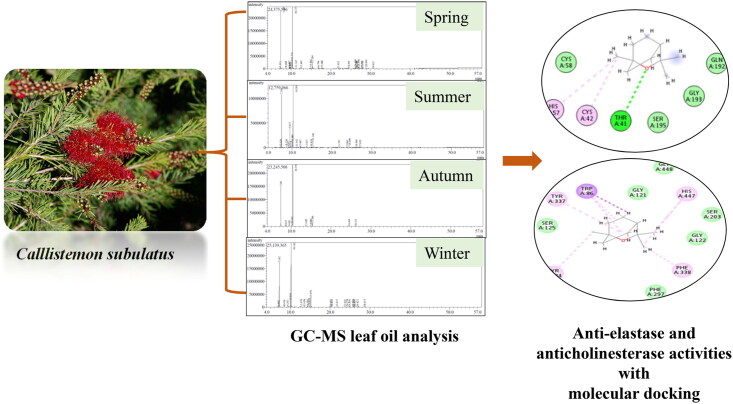

## Introduction

Historically, plants have recorded a great importance for utilisation in different health purposes in various countries such as Mesopotamia, Egypt, Greek, and Islamic cultures^1–3^. Medicinal plants continuously offer new and promising lead compounds with various pharmacological effects in different disorders such as ageing, Alzheimer, cancer, malaria, nephritis and hepatitis^4–6^.

*Callistemon* (Myrtaceae) is mainly found in tropic and sub-tropic areas around the world. It contains more than 35 species of shrubs or small trees with evergreen leaves and flower spikes that have a traditional bottle brush shape^7^. Different parts of the *Callistemon* plant have traditionally been used as anthelmintic^8^, anti-inflammatory^9^, antiviral, antimicrobial, pleasant-smelling tea substitute and in ornamental horticulture^10^.

*Callistemon subulatus* is a species native to south eastern Australia and recently cultivated in Egypt for ornamental purposes^11^. It is a short shrub with densely arranged leaves and dark crimson filaments. It flowers in late spring and early summer. Previous studies have shown that the leaves are rich source of volatile oils^11^ and polyphenolics^12^, and showed the ability of *C. subulatus* to be cytotoxic^11^, antimicrobial^13^, anti-nociceptive, anti-inflammatory, antidiarrheal, and antioxidant^12^^,^^14^.

The skin ageing symptoms are represented by loss of integrity and the appearance of wrinkles in the skin caused by a gradual decrease of skin proteins’ production such as elastin, and their increased damage by the action of degrading enzymes such as elastase, these enzymes are upregulated by different impacts as ageing, UV radiation and reactive oxygen species^15^^,^^16^. Elastase overexpression also contribute to other inflammatory conditions such as rheumatoid arthritis, cyclic neutropenia, sepsis, cystic fibrosis, asthma and atherosclerosis^17^.

Alzheimer disease is a neurodegenerative disease associated with disturbance in cholinergic transmission, resulting in memory loss, behavioural changes and cognitive impairment^18^. Acetylcholinesterase (AChE) inhibitors are one of the most effective therapeutics for Alzheimer disease, as they alter the excess of synaptic AChE and increase acetylcholine levels^19^.

Essential oils, containing compounds of relatively small molecular size and high lipophilicity, have a high ability to be absorbed through skin layers and to penetrate blood brain barrier^20–22^, which could be an effective approach for skin repair and for management of brain disorders. In addition, several studies reported the anti-elastase and anti- acetylcholinesterase activities of several plants belonging to the genus *Callistemon* and their metabolites^23–25^. This directs the search for other *Callistemon* species with potential towards these enzymes.

Therefore, the aim of the work is to identify the chemical constituents of *C. subulatus* essential oils collected in the four seasons of the year by GC-MS analysis to reveal their seasonal variation and to evaluate the ability of the highest yield oil (winter oil) to inhibit both elastase and AChE enzymes to unravel its potential as anti-ageing supplement. In addition, molecular docking studies for the major oil constituents were performed on these enzymes to support the observed results. It should be noted that the seasonal variation and anti-ageing evaluation of *C. subulatus* essential oil was carried out for the first time herein.

## Materials and methods

### Plant material and isolation of oil

Fresh leaves of *Callistemon subulatus* were harvested during the four seasons, in January, April, June and October 2021 (500 g each), from El-Orman Garden in Giza, Egypt. The plant was identified by Mrs. Treize Labib, the taxonomist at El-Orman Botanical Garden. The voucher specimen, PHG-P-CS-416, was placed in the Herbarium of Pharmacognosy Department, faculty of Pharmacy, Ain Shams University. Leaves collected in each season were hydro-distilled by a Clevenger apparatus for 6 h. The collected oils were then refrigerated and chemically evaluated by GC/MS analysis.

### Gas Chromatography-Mass spectrometry (GC/MS) analysis

Analysis was performed by TRACE GC-Ultra gas chromatography, conjugated with thermal mass spectrometer (MS) detector (THERMO Scientific Corp., USA, and ISQ Single Quadrupole Mass Spectrometer). It was supplied with DB-5 column (30 m × 0.25 mm × 0.25 μm film thickness), a carrier gas was helium (flow rate of 1.0 mL/min). The injector temperature was 250 °C with split ratio of 1 to 15, about 0.2 μL of diluted oil samples (1: 10 in hexane) were injected. The oven temperature program was 80 °C for 2 min; then rising of 5.0 °C/min till 300 °C with hold for 5 min. Electron Ionisation (EI) MS has a spectral range of 20 to 500 *m/z.* The resulting peaks were assigned by matching retention indices (RI) with those found in the literature^21^^,^^26–28^ and online libraries such as NIST.

### Evaluation of antiaging activities

#### Anti-elastase activity

The elastase inhibitor activity of the tested oil was screened using EnzChek® Elastase Assay Kit (E-12056), including *N*-methoxysuccinyl-Ala-Ala-Pro-Val-chloromethyl ketone, as a standard elastase inhibitor. The assay was done according to the provided manual. The concentration of oil causing 50% elastase inhibition (IC_50_) was calculated from the graph correlating the concentration and % of enzyme inhibition.

#### Anti-cholinesterase activity

The evaluation of anticholinesterase activity of collected oil of *C. subulatus* had been done by Bio-vision AChE inhibitor screening kit (Catalog # K197-100). The kit contained an inhibitor control donepezil (20 µM), colorimetric substrate for the reaction; Acetylthiocholine iodide and (5,5′-dithio-bis(2-nitrobenzoic) acid (DTNB) for assessment of AChE activity. The graph correlating the oil concentration and the percentage of AChE inhibition gives IC_50_ value of the oil.

### Molecular docking study

The major identified compounds by GC-MS analysis in the highest yield (winter) oil of *C. subulatus* leaves (eucalyptol, α-pinene and α-phellandrene) were docked into the active sites of elastase and acetylcholinestrase (AChE) enzymes. The software implicated was autodock Vina. Docking was done as per reported methodology^29^^,^^30^. The crystal structures of both enzymes co-crystallized with ligands were downloaded from the protein data bank with PDB-IDs as follows (6qeo for elastase and 7d9o for acetylcholinesterase). The compound structures were drawn using ChemDraw Ultra 8.0 and Amber12: EHT force field was applied for energy minimisation. Conformers of lowest energies are saved by the program. Docking validation was carried out by re-docking the co-crystallized ligands into the active sites of their respective enzymes. Following revealing of the active sites, the identified compounds of the oil were docked separately into them and their docking was analysed by Biovia Discovery Studio visualiser to show their binding interaction diagrams.

## Result and discussion

### Chemical composition of leaf essential oil and seasonal variation

**Figure 1.**
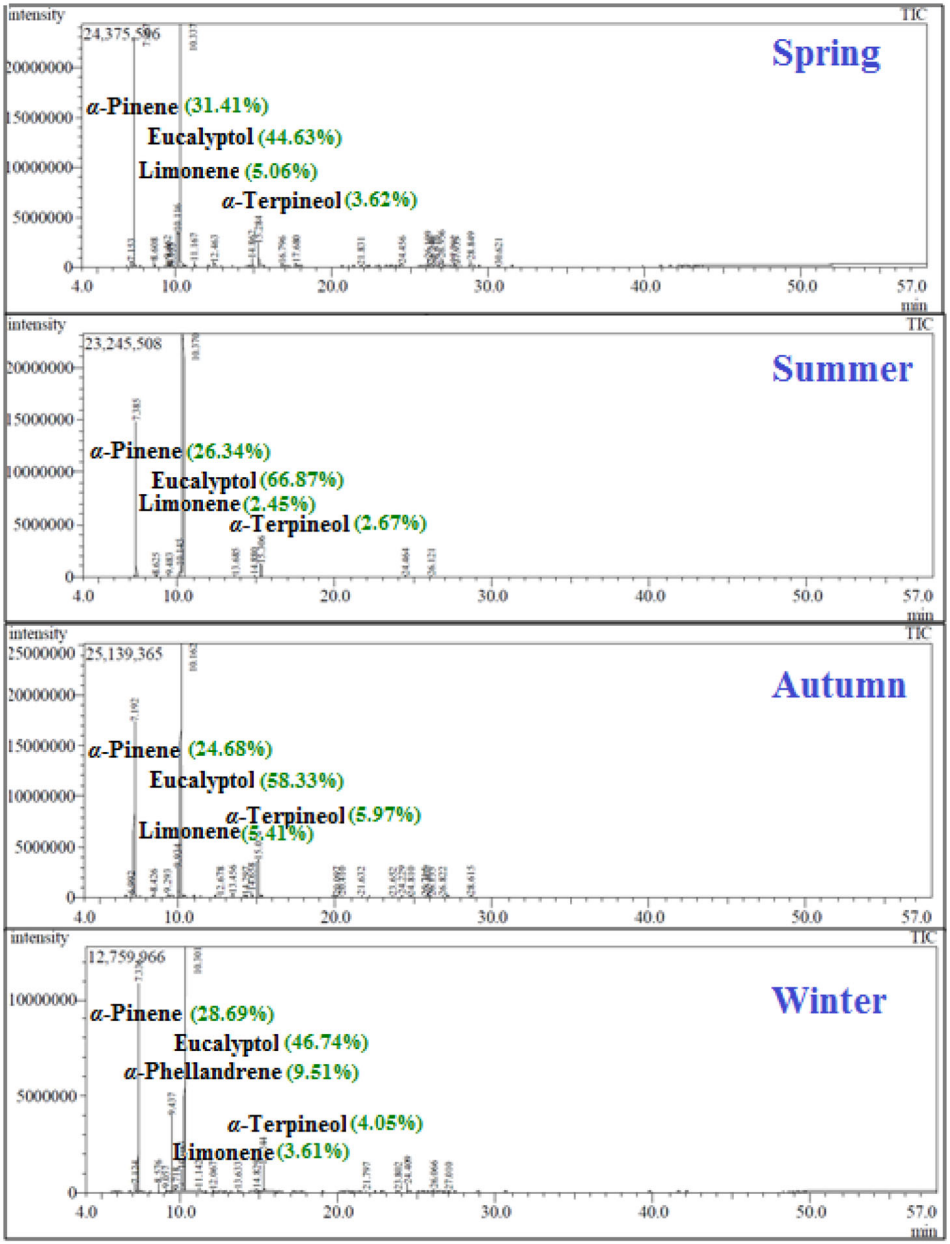
GC chromatograms of *C. subulatus* leaves essential oils showing their seasonal variation.

The GC/MS analysis showed the presence of 31 compounds in *C. subulatus* leaf essential oils, the spring oil contained the largest number of compounds; 23 compounds (98.96%), followed by autumn; 22 compounds (100%), then winter; 18 compounds (99.93%), and finally summer has only has 10 compounds (99.99%), so the highest chemical diversity appeared in spring and autumn. The chemical constituents of the oil and the yield in each season have been illustrated in (Table 1, Figure 1).

**Table 1.** GC-MS analysis of *C. subulatus* leaf oils in the four seasons.

No.	Compound name	Retention time	Molecular formula	RI _exp_	RI _lit_	Relative abundance (%)			
						Spring	Summer	Autumn	Winter
1.	*α*-Thujene	7.155	C_10_H_16_	908	906	0.64	–	0.25	0.98
2.	***α*-Pinene**	**7.365**	**C_10_H_16_**	**917**	**917**	**31.41**	**26.34**	**24.68**	**28.69**
3.	*β*-Pinene	8.576	C_10_H_16_	962	963	0.72	0.25	0.52	0.97
4.	*β*-Myrcene	9.057	C_10_H_16_	978	978	–	–	–	0.45
5.	*α*-Phellandrene	9.460	C_10_H_16_	993	994	1.03	0.32	0.64	9.51
6.	2-Carene	9.635	C_10_H_16_	999	1000	0.10	–	–	–
7.	3-Carene	9.755	C_10_H_16_	1003	1004	0.17	–	–	0.38
8.	Limonene	10.115	C_10_H_16_	1016	1018	5.06	2.45	5.41	3.61
9.	**Eucalyptol**	**10.335**	**C_10_H_18_O**	**1022**	**1022**	**44.63**	**66.87**	**58.33**	**46.74**
10.	*γ*-Terpinene	11.165	C_10_H_16_	1049	1049	0.76	–	–	0.58
11.	*α*-Terpinolene	12.067	C_10_H_16_	1090	1085	0.74	–	–	0.34
12.	Linalool	12.678	C_10_H_18_O	1113	1113	–	–	0.14	–
13.	Pinocarveol	13.633	C_10_H_16_O	1138	1138	–	0.23	0.45	0.42
14.	Borneol	14.297	C_10_H_18_O	1166	1166	–	–	0.16	–
15.	Terpinen-4-ol	14.860	C_10_H_18_O	1168	1168	1.27	0.43	0.89	0.60
16.	*α-*Terpineol	15.285	C_10_H_18_O	1181	1181	3.62	2.67	5.97	4.05
17.	*β*-Citral	16.795	C_10_H_16_O	1232	1232	0.63	–	–	–
18.	*α*-Citral	17.680	C_10_H_16_O	1263	1267	1.27	–	–	–
19.	Eugenol	20.097	C_10_H_12_O_2_	1366	1366	–	–	0.20	–
20.	Geranyl acetate	20.410	C_12_H_20_O_2_	1372	1377	–	–	0.15	–
21.	Caryophyllene	21.797	C_15_H_24_	1409	1409	0.27	–	0.18	0.26
22.	Ledene	23.652	C_15_H_24_	1500	1495	–	–	0.12	0.26
23.	*δ*-Cadinene	24.455	C_15_H_24_	1512	1515	0.56	0.22	0.34	1.20
24.	flavesone	24.810	C_14_H_20_O_4_	1547	1546	–	–	0.17	–
25.	Spathulenol	26.066	C_15_H_24_O	1577	1577	1.39	–	0.14	0.50
26.	Viridiflorol	26.121	C_15_H_26_O	1586	1590	0.61	0.21	0.57	–
27.	Guaiol	26.610	C_15_H_26_O	1596	1600	0.44	–	0.18	–
28.	Caryophyllene oxide	27.010	C_15_H_24_O	1613	1615	2.34	–	0.20	0.39
29.	Guaia-3,9-diene-11-ol	27.790	C_15_H_24_O	1647	1649	0.57	–	–	–
30.	Neointermedeol	27.935	C_15_H_26_O	1653	1656	0.39	–	–	–
31.	Germacrone	28.850	C_15_H_22_O	1693	1696	1.40	–	0.32	–
	Monoterpene hydrocarbons					40.66	29.36	31.5	45.51
	Oxygenated monoterpenes					51.72	70.2	66.29	51.81
	Sesquiterpene hydrocarbons					0.83	0.22	0.64	1.72
	Oxygenated Sesquiterpene					7.14	0.21	1.58	0.89
	Total identified (%)					98.96	99.99	100	99.93
	Yield (mL/100 gm)					0.17	0.26	0.34	0.53

The major oil components are written in bold.

**Figure 2.**
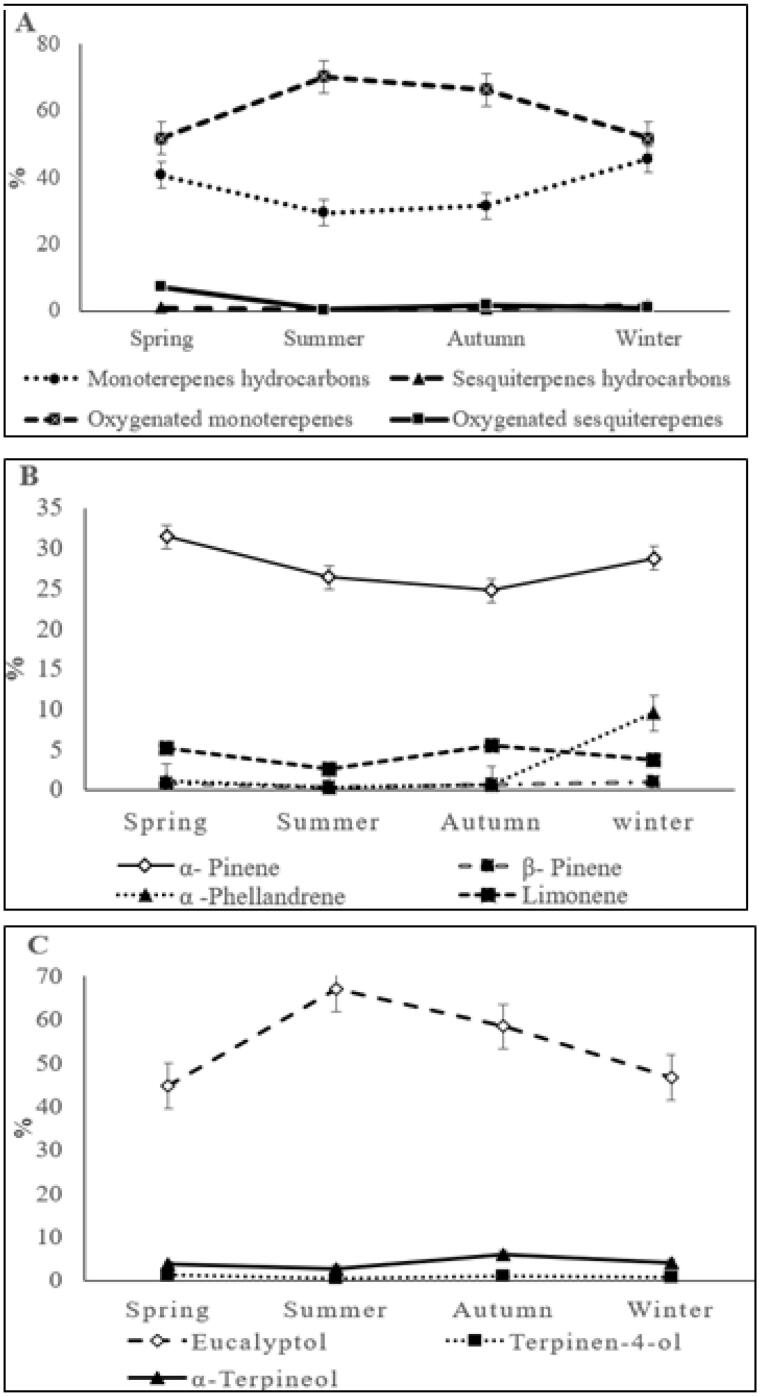
Seasonal variation in the major compounds of *C. subulatus* leaves oil, showing seasonal variation of the total classes of compounds (A), the four major monoterpenes hydrocarbons (B) and the three major oxygenated monoterpenes (C).

Of the 31 compounds identified, 20 are monoterpenes and 11 are sesquiterpenes, the monoterpenes also found in higher relative abundance than sesquiterpenes. In the summer oil, the monoterpenes (99.56%) are up to 230 times of the sesquiterpenes (0.43%), but this ratio in the spring oil decreased to only 11 times since the abundance of sesquiterpenes in this oil reached 8% (Figure 2).

The main components of the plant essential oils are terpene hydrocarbons and oxygenated terpenes, terpenoids^31^. In *C. subulatus* essential oil, the highest content of monoterpene hydrocarbons (MTHC) was in winter (45.51%) while it was the lowest in the summer (29.36%), it has been previously explained that these MTHC compounds are light, small and thermolabile so they are more fixed to leaves in less hot and more humid climates, decreasing their volatilisation^32^. The identified oxygenated terpenes in *C. subulatus* oil were 18 compounds, which are monoterpenoids and sesquiterpenoids. Ten monoterpenoids identified in *C. subulatus* oil that were equal to the number of monoterpene hydrocarbons, the number of identified sesquiterpenoids was 8 while only 3 sesquiterpenes hydrocarbons were found. The relative abundances of monoterpenoids in all seasons was more than that of monoterpene hydrocarbons, they reached more than doubled in summer and autumn. While the sesquiterpenoids were more abundant than sesquiterpenes hydrocarbons in both spring and autumn, they were less abundant in winter and about the same in summer.

The major compound in all-seasons oils was eucalyptol (1,8 cineol); oxygenated monoterpene, and in summer it exceeds 70% of the entire oil content, this is consistent with what has been published that 1,8 cineol has been the main terpene in most of chemical analysed *Callistemon* oils^33^. Second major compound was α-pinene, a monoterpene hydrocarbon, which accounts for about a third of spring and winter oils. Then followed by α-phellandrene (9.51%) in winter; α-terpineol in autumn and summer (5.97 and 2.67%, respectively); and limonene in spring (5.6%).

Our results regarding the chemical composition of *C. subulatus* came in agreement with previously published data. Brophy et al. reported that eucalyptol was the most abundant terpene identified in the leaves of Australian *C. subualtus*^34^, representing 61.3%. Also, it was reported to be the major constituents of Egyptian chemotype (48.97%)^11^. α-Pinene (31.59%), α-terpineol (7.84%) and limonene (5.27%) were reported in C. subulatus oil, in agreement with the autumn and summer essential oils of the current study. However, *α*-pinene (47.06%) was the major in the *C. subulatus* oil in another study, then eucalyptol (16.13%) and α-terpineol (15.27%)^13^.

### Assessment of antiaging activities

#### Anti-elastase activity

Elastin is a skin protein that plays a vital role in maintaining the healthy appearance and integrity of the skin^15^. Activation of elastase by ageing or exposure to UV rays and free radicals leads to the destruction of cutaneous elastin and thus loss of skin elasticity. The *C. subulatus* leaf oil of winter showed an inhibition to elastase activity with IC_50_ value of 1.05 ± 0.05 µg/mL, compared to the standard N-methoxysuccinyl-Ala-Ala-Pro-Val-chloromethyl ketone (IC_50_ = 0.55 ± 0.03 µg/mL). The essential oil of *Illicium anisatum* containing eucalyptol as a major component (36.7%) was able to inhibit elastase (IC_50_ = 1.79 mg/mL)^35^. Eucalyptol was one of the most abundant terpenes (11.82%) in *Mentha viridis* essential oil that showed elastase inhibition by IC_50_ = 114.24 ± 1.22 µg/mL^36^.

#### Anti-cholinesterase activity

The inhibition of AChE was introduced as therapeutic strategy for Alzheimer management via prevention of acetylcholine degradation causing enhancement in cholinergic transmission^37^. The winter leaf oil showed AChE inhibitory activity with IC_50_ value of 0.208 ± 0.011 µg/mL, this compared to the standard donepezil (IC_50_ = 0.045 ± 0.002 µg/mL). Other *Callistemon* species showed potential for neuroprotection through AChE inhibitory activity, as the essential oil of *C. citrinus* aerial part, containing eucalyptol (55.40%) as the major component, showed a strong AChE inhibitory with an IC_50_ value of 6.335 μg/mL, slightly lower than that of galanthamine (IC_50_ = 6.652 µg/mL)^25^. Commercially available 1,8-cineole and *α*-pinene showed AChE inhibitory activities with IC_50_ values of 0.015 and 0.022 mg/mL, respectively that were 0.017–0.025 fold of galantamine^38^. The major components of *Salvia lavandulaefolia* essential oil: eucalyptol and α-pinene, showed dose-dependent inhibition of AChE with IC_50_ values of 0.05, and 0.06 mg/mL, respectively^39^.

### Molecular docking study

**Figure 3.**
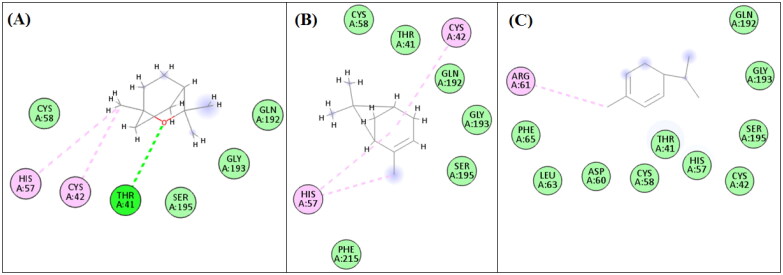
2D-Binding diagrams of eucalyptol (A), α-pinene (B) and α-phellandrene (C) on the active site of elastase enzyme.

Molecular docking has been indispensable in recent researches, to display binding affinities of bioactive molecules in the target enzymes and to validate their proposed mechanism of bioactivities^40^^,^^41^. As shown in Figure 3, eucalyptol revealed hydrogen bonding with ThR A:41 amino acid moiety present in the active site of elastase enzyme, in addition to two alkyl and Pi-alkyl interactions with HIS A:57 and CYS A:42 as well as van der Waals interactions with CYS A:58, SER A:195, GLY A:193 and GLN A:192 amino acids. Similarly, α-pinene revealed three alkyl and Pi-alkyl interactions with HIS A:57 and CYS A:42 amino acids and numerous van der Waals interactions. This was also observed to a lower extent in α-phellandrene binding interactions.

**Figure 4.**
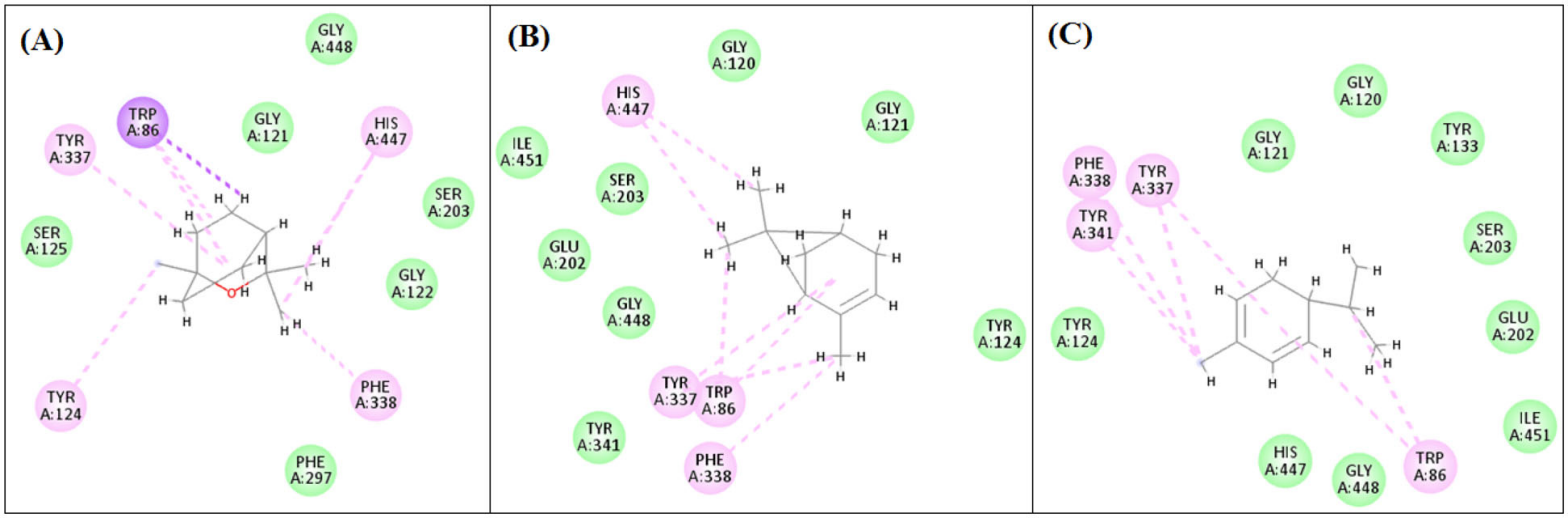
2D-Binding diagrams of eucalyptol (A), α-pinene (B) and α-phellandrene (C) on the active site of acetylcholinestrase enzyme.

Regarding acetylcholinesterase enzyme (Figure 4), eucalyptol showed one Pi-Sigma interaction with TRP A:86, six Pi-Alkyl interactions with HIS A: 447, TRP A: 86, TYR A:337, TYR A:124, PHE A:338, and van der Waals interactions. α-Pinene showed seven Pi-alkyl interactions with HIS A:447, TYR A:337, TRP A:86, PHE A:338, in addition to van der Waals interactions. α-Phellandrene showed many van der Waals interactions and six Pi-alkyl interactions with TYR A:337, TYR A:341, PHE A:338, and TRP A:86 amino acid moieties.

Eugenol showed the best results revealing good binding affinities to both enzymes and lowest binding energies as demonstrated in Table 2.

**Table 2.** Docking binding energies of major identified compounds of *C. subulatus* winter oil with elastase and acetylcholinesterase enzymes.

Compound	Binding energy (Kcal/mol)	
	Elastase	Acetylcholinesterase
Eucalyptol	−8.6	−9.7
α-Pinene	−7.7	−7.9
α-Phellandrene	−7.1	−7.3

## Conclusions

The essential oil of *C. subulatus* leaves is rich in monoterpenes, especially monoterpenoids. On the contrary, sesquiterpenes are very low in the oil and their concentration varies according to the harvest season, being much lower in summer oil while reaching their highest concentration in spring oil. The essential oil of *C. subulatus* has shown antiaging prospects, so further research is needed to discover more specifically the constituent responsible for this activity and to be applied in skin care formulations and as adjuvant in Alzheimer therapy, enhancing memory and concentration.
